# Neuromagnetism “On the Cheap”: Evaluating a Combined Cylindrical Shield and Partial-Coverage OPM-MEG System for Detecting Sensorimotor Responses in Humans

**DOI:** 10.3390/s26103131

**Published:** 2026-05-15

**Authors:** Lyam M. Bailey, Clara Knox, Timothy Bardouille

**Affiliations:** 1 Diagnostic & Interventional Radiology, Hospital for Sick Children, Toronto, ON M5G 0A4, Canada; lyam.bailey@sickkids.ca; 2 Department of Physics & Atmospheric Science, Dalhousie University, Halifax, NS B3H 4R2, Canada

**Keywords:** OPM, MEG, MNS

## Abstract

Background: Optically pumped magnetometers (OPMs) have emerged as a promising technology for neuromagnetic recording in humans. Current state-of-the-art OPM systems are housed in large magnetically shielded rooms to reduce external electromagnetic noise and typically comprise sensor arrays covering the entire head. Such systems are extremely costly to purchase and install, and take up large amounts of physical space, which limits the accessibility of this technology to research groups with limited funding. Here we sought to evaluate the utility of a more accessible “starter” OPM system comprising a small cylindrical mu-metal shield and partial sensor coverage. Methods: Twelve participants underwent right-sided median nerve stimulation (MNS) intended to elicit ubiquitous sensorimotor responses: somatosensory-evoked fields (SEFs, comprising N20m, P35m and P60m components) and event-related (de)synchronization (ERD/ERS) of oscillatory neuronal rhythms in the mu and beta frequency ranges. Results: Following MNS, we observed robust N20m and P60m peaks, as well as the expected mu ERD and beta ERS effects. Moreover, these responses could be localized to expected cortical generators. However, we observed markedly lower SNR than that seen in state-of-the-art systems. We make recommendations for further improvements to this system and others like it.

## 1. Introduction

Over the past decade, optically pumped magnetometers (OPMs) have emerged as a promising alternative to conventional systems used for magnetoencephalography (MEG)—a non-invasive imaging technique for neuroelectrophysiological recording in humans. MEG is a valuable tool for neuroscientists because it allows us to record millisecond-to-millisecond fluctuations in neuronal activity (e.g., over the course of rapidly changing experimental conditions), and offers relatively high (2–5 mm) spatial resolution on the cortical surface [1,2]. Conventional cryogenic MEG systems, which rely on superconducting quantum interference devices (SQUIDs), are relatively inaccessible for most research groups because they are expensive, require housing in large magnetically shielded rooms, and demand a constant supply of liquid helium to keep their sensors operational. Moreover, SQUID-MEG is extremely sensitive to participant movement; this limits its utility in individuals who have difficulty remaining still throughout a scan (e.g., children and some clinical populations). By contrast, OPM-MEG systems are less costly to purchase, do not require liquid helium, and are relatively insensitive to movement [1], while also providing comparable or superior sensitivity to human neuronal activity [3,4,5,6].

OPM systems are also modular-sensors that can be purchased and utilized individually rather than in fixed arrays. The lack of a cryogenic dewar and fixed sensor array reduces space requirements and permits smaller shields, e.g., [7,8]. Thus, it is possible to record neural activity at a reduced cost, as compared to a whole-head system in a large MSR–providing a new “on ramp” for researchers interested in adding MEG to their program. Moreover, MSRs inherently limit the mobility of OPM-MEG and take up large amounts of physical space; smaller scanners may facilitate translation to clinical and rural environments. In this vein, early work has demonstrated MEG recordings with cylindrical shields approximately the size of a hospital bed [3,8,9]. Unfortunately, there are substantial technical challenges to recording OPM with fewer channels and when the shield is close to the sensor array. Here, we report on human neuromagnetic recordings from such a system with the goal of providing an honest quantification of what operators can expect to achieve with low-channel-count OPM recordings in a commercially available cylindrical shield.

**Figure 1:**
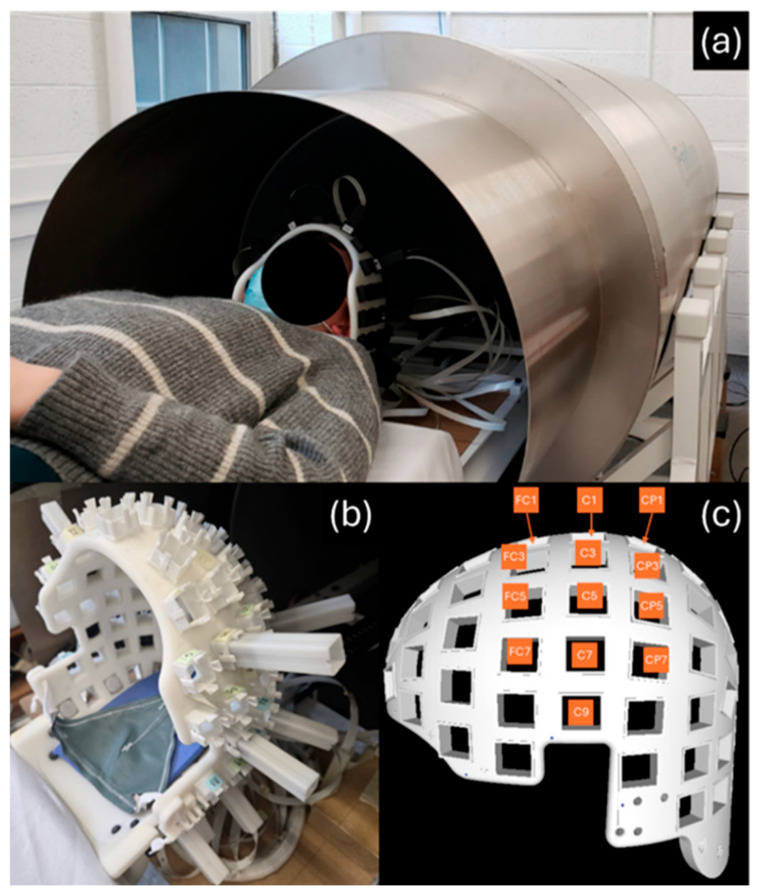
The cylindrical shield, partial coverage OPM system. (**a**) A participant ready to be inserted into the shield. (**b**) The rigid helmet into which sensors may be inserted. (**c**) The sensor configuration used in this study.

The system evaluated here, previously described by Bardouille et al. [7] comprises a dual-wall cylindrical mu-metal shield, mounted on a wooden cradle, and a rigid helmet in which OPM sensors can be positioned against a participant’s head (Figure 1a,b). Active field compensation for this system is under development and therefore was not implemented at the time of this study. The system also comprises sixteen single-axis OPM sensors which can be flexibly arranged to overlay a given area of the head. In this work we arranged sensors over left sensorimotor areas, with a view to capture neuromagnetic signals elicited by contralateral (right-sided) stimulation (Figure 1c). Sensitivity to these areas of interest was confirmed via simulation.

Median nerve stimulation (MNS) was chosen for this study on system performance because it reliably elicits neuromagnetic biomarkers of sensorimotor processing. These cortical responses are so reliable that MNS is used as part of pre-surgical mapping paradigms, where results in a single participant are critical [10]. In particular, MNS elicits a somatosensory-evoked field (SEF) complex that is characterized by peaks around 20, 35, and 60 ms following stimulation (labeled N20m, P35m, and P60m respectively). Evidence from MEG indicates that the SEF originates in primary somatosensory cortex, in Brodmann area 3b (i.e., the posterior bank of the central sulcus) contralateral to the side of stimulation [11,12,13,14]. With a sufficiently long inter-stimulus interval (ISI), MNS also elicits neuronal rhythm changes in the mu (8–15 Hz) and beta (15–30 Hz) frequency ranges. These changes (also revealed in transient finger movement) entail an immediate reduction in mu and beta power, relative to a preceding baseline period (event-related desynchronization; ERD); this is followed by a return to baseline levels in the mu range and a “rebound” (event-related synchronization; ERS) in beta power [15,16,17,18,19]. In terms of their underlying cortical generators, mu and beta ERD are typically localized to central-to-posterior sensorimotor cortex contralateral to movement or stimulation, while the beta rebound tends to appear more anteriorly [15,18]. SEFs and oscillatory rhythm changes are ubiquitous in MEG research and can be detected by contemporary OPM systems housed in MSRs [6,20,21,22,23]. As such, they provide a useful benchmark by which to validate our system.

We evaluated the ability of our OPM-MEG system to detect these ubiquitous sensorimotor responses following MNS. Besides detecting these responses at the sensor level, an important ability for any MEG system is source estimation. Characterizing this ability is particularly salient for evaluating our system, considering that low sensor count and partial head coverage might impede sensitivity. As such, we assessed whether SEF and oscillatory responses detected at the sensor level could be localized to areas of contralateral sensorimotor cortex consistent with the wider literature. Much previous work with this [7] and similar [3,4,8] systems has relied on point-like source estimation using the equivalent current dipole (ECD). However, we chose to apply magnetic source mapping using minimum norm estimation (MNE). This type of approach is commonplace in OPM research, e.g., [6,24,25] and has been demonstrated in systems with low channel counts e.g., [26]; therefore, we used this approach in the interests of better contextualizing our system performance with common research practices. This also allowed us to compare our experimental findings to sensitivity maps generated via simulation.

Participants completed two right-sided MNS protocols (short and long ISI) inside the scanner. We expected that MNS would elicit SEFs and typical modulations in the mu and beta ranges (mu and beta ERD followed by beta ERS). Moreover, we expected that distributed source modeling would localize these responses to contralateral sensorimotor cortex. More precisely: SEFs and mu/beta ERD should be maximal in the central sulcus and postcentral gyrus. Meanwhile, beta ERS should be maximal more anteriorly, in the precentral gyrus. The above predictions reflected important benchmarks for evaluating the utility of our “starter” OPM system for human neural recording. More broadly, we sought to contextualize this system in the wider field by quantifying the signal-to-noise ratio (SNR) of our sensor- and source-level measurements, for comparison with SNRs reported by studies using more conventional (but less accessible) systems.

## 2. Materials and Methods

Twelve volunteers (6 female, aged 19–36, mean age 24.17) were recruited through on-campus advertising at Dalhousie University and were compensated with $25 CAD for their time. All participants were right-handed as determined by the Edinburgh Handedness Inventory [27], neurologically healthy (no previous stroke or brain lesion), were not taking medication for a neurological or psychiatric disorder, and were free of metal implants, hearings aids, and non-removable metal piercings. During recruitment and informed consent, potential participants were advised that individuals with claustrophobia might experience anxiety inside the shield, and that they should consider this before agreeing to participate. Three subjects had non-removable wire dental retainers; these were de-magnetized immediately prior to OPM scanning using an audio/video tape eraser (RadioShack, Canada). All procedures were approved by the Dalhousie University Research Ethics Board (REB #2021-5586).

The following description of the cylindrical mu-metal shield and OPM sensor array have been adapted from Bardouille et al. [7]. The cylindrical shield (Figure 1a) comprises inner and outer layers of mu-metal, with 43 cm and 48 cm radii and axial lengths of 1.9 m and 2.1 m, respectively (American Magnetics, Oak Ridge, TN, USA). The open end of the cylinder (for participant insertion) includes a 45-cm-long reducer to a radius of 38 cm. At the other end of the cylinder, the inner and outer shields are closed with friction-fitted end caps. The thickness of the 5 mu-metal components is 1.8 mm. A smaller-diameter polymer cylinder includes coils for nulling the homogenous field along each cardinal axis (i.e., active shielding; not used in this study). The cylinder is mounted on a wooden cradle, and participants are supported (lying supine) on a retractable wooden bed, on a non-metal rail system which allows the participant bed to be inserted head-first until the helmet is approximately 1.65 m into the cylinder. The shield end at the participant’s feet always remains open (i.e., there is an end cap at the participant’s head end only). Once inserted into the shield, the participant’s head is situated about 60 cm from the end cap. A small bore in the end cap (enabling external projection of visual stimuli; not used here) allows air flow through the cylinder (no heating or cooling is required). Ambient lighting from the surrounding room allows participants to see inside the cylinder.

Sixteen FieldLine v2 single-axis OPM sensors (Fieldline Inc., Boulder, CO, USA), with less than 20 fT/√Hz sensitivity and a bandwidth of 0–150 Hz, were used in this study. Data were acquired with the sensors in closed loop mode. Device control and 24-bit data acquisition were managed via vendor-supplied v2 electronics chassis, and a personal computer was running vendor-supplied acquisition software (Recorder, v1.6.7) on the Windows 11 operating system. A rigid helmet for mounting OPM sensors is fixed to the end of the wooden bed (Figure 1b). OPM sensors are inserted into slots in the helmet at variable depths, such that they can be positioned against a participant’s head. The OPM sensor positions with respect to the helmet can be precisely known by measuring the depth of the sensor in each slot. The orientation is precisely defined for each slot based on the helmet manufacture, independent of depth. The sensors are silent and operate at a maximum temperature of ~30 °C, meaning they can be placed safely and comfortably against the head. Three of the 16 sensors were arranged in orthogonal orientations and fixed in place approximately 35–40 cm superior, posterior and lateral (participant left) to the top of the helmet (i.e., Cz location) to be used as reference sensors; the remaining 13 were inserted into the helmet for participant scans. These 13 sensors were arranged in a left-sided configuration (Figure 1c) over sites where sensorimotor responses ought to be maximal (FC1, C1, CP1, FC3, C3, CP3, FC5, C5, CP5, FC7, C7, CP7, C9). Sensor placement was determined manually based on prior experience (with other MEG systems and with the SEF topography previously observed this system), as well as literature review. A post hoc confirmation of the validity of this layout was performed using sensitivity maps, as described below.

Each participant completed informed consent and was given a brief tour of the OPM system before beginning the experiment and encouraged to ask any questions or express any concerns they had before entering the scanner. Prior to the MEG recording, we collected a 3-D digital image of the participant’s head shape using an optical imaging system (EinScan H2, Shining 3D) to register the MEG data to a template MRI. Colored markers were placed on the fiducial points (nasion, left and right preauricular points) to facilitate manual identification in the resultant images. Then, the participant was instructed to lie supine on the scanner bed with their head positioned inside the OPM helmet. Layers of foam padding were used to cushion the back of the participant’s head against the helmet and, when necessary, additional padding was inserted to ensure that the participant was as comfortable as possible and to reduce head movement inside the helmet. Thirteen OPM sensors were inserted into the helmet such that they made direct contact with the participant’s head. We collected a second 3-D digital image of the front of the participant’s head positioned inside the OPM helmet to register the participant’s head to the MEG sensors. Participants completed four scans in a single session, always in the same order: two MNS protocols, a cued button-press task, and a resting-state scan in which they were instructed to lie still with their eyes closed for approximately 5 min (data from the button-press and resting scan are not reported here). Each participant was inside the scanner for approximately 30 min. Between tasks, a member of the research team communicated verbally with the participant to inform them of the upcoming task, and to ensure that they were still comfortable inside the scanner. Following the experiment, the depth of each sensor in the helmet was manually recorded. After exiting the scanner, each participant was asked about their experience. None reported excessive discomfort.

Prior to scanning we fitted each participant with moistened stimulation electrodes housed in a plastic casing, positioned such that the electrodes aligned with the median nerve on their right inner wrist. The electrodes were connected via an insulated cable to a DS7A Constant Current Stimulator (Digitimer, Welwyn Garden City, UK). Stimulation was delivered at 250 V in single 500 µs pulses. Stimulation current was the minimum current required to elicit a small twitch of the right thumb (determined for each participant individually). Participants completed two MNS protocols—labeled “short MNS” and “long MNS”—lasting approximately 5 and 7 min respectively. In the first (short MNS) protocol, participants received 300 stimulations delivered 1–1.4 s apart. In the second (long MNS), participants received 80 stimulations delivered 5.6–7.6 s apart. Data from the short and long MNS protocols were used to investigate SEF responses and oscillatory modulations respectively, since longer inter-stimulus intervals (ISIs) are needed to properly quantify beta ERS [28,29,30].

Unless otherwise stated, all analyses were performed in the Python (v3.14.3) environment using functions from the MNE [31] and Open3D [32] libraries, and custom scripting. We relied on the FreeSurfer template brain for source localization; this required co-registration of participants’ head digitization and sensor positions to the template head model. We first conducted surface reconstruction, and computed a boundary element model BEM [33], from the T1 template image using FreeSurfer’s recon-all algorithm. We then applied the following procedure for each participant.

The two 3D images of the participant’s head, as well as a vendor-supplied computer-aided design image of the OPM helmet, were manually aligned using MeshLab. The positions of the fiducial markers were manually determined using the 3D image acquired outside of the helmet. These locations defined the head coordinate frame (*x*-axis through the preauricular points and *y*-axis through the nasion). Sensor data were registered to this coordinate frame, as follows. Sensor positions and orientations in the helmet coordinate frame were determined based on known locations of each sensor slot and manually recorded sensor depths. Following this, the 3-D image of the head in the helmet was registered to the head-only image using the iterative closest point (ICP) algorithm in Meshlab. We manually identified four points that were common to the head-only and head-in-helmet images (e.g., the three fiducial markers and the tip of the nose); these points were used for ICP. Finally, a vendor-supplied helmet shape was aligned to the sensor slots of the head in helmet 3-D image, using the same ICP procedure. The two resulting transformation matrices were combined with the sensor positions in the helmet coordinate frame to provide the sensor positions in the head coordinate frame. MRI to head registration proceeded as follows. The template anatomical T1 MRI was manually scaled and translated for optimal fit to each individual head, using a visual matching process implemented via the MNE co-registration graphic user interface. Importantly, the template BEM and brain surface were also scaled alongside the T1 image; this allowed us to compute a forward model and source-space grid along the (scaled) cortical surface for each participant. This approach was implemented because T1 MRIs were not available from the participants.

Raw OPM data was notch filtered to remove line noise and its 1st and 2nd order harmonics (60, 120, and 180 Hz) and then band-pass filtered between 3 and 150 Hz. We removed channels with remaining high-frequency spikes; these were identified by computing a z-scored spectral amplitude per channel in the 120–145 Hz band. We removed channels with z-score > 2.0. We also visually inspected the raw data to identify and remove any additional bad channels (e.g., with repeating artifacts such as extreme amplitude spikes). Signals that were strongly correlated with the three reference sensors were removed using reference array regression as described in Bardouille et al. [7]. In brief, we performed a multivariate linear regression which treated each reference sensor as a dependent variable, and each channel as an independent variable. The residuals from each channel were taken as reference-adjusted time courses for that channel and carried through to subsequent analyses. We then performed homogenous field correction (HFC; order = 1) to attenuate noise in the raw data arising from ambient magnetic fields with a consistent and homogenous spatial pattern over time [7,34].

The filtered, re-referenced, and HFC-corrected data were segmented into epochs according to event timing in each scan. Epochs from the short MNS data were 0.7 s in length (−0.2 to +0.5 s relative to stimulation onset); long MNS epochs were 6.4 s (−1.2 to +5.2 s). Epochs were baseline-corrected by subtracting the mean baseline signal from the entire epoch; baseline periods for each dataset were −0.2 to −0.1 s (short ISI), and −1.0 to −0.5 s (long MNS). We visually inspected the epoched data to identify and remove artifacts (we rejected 1.39% and 6.35% of epochs from the short MNS and long MNS data respectively). For the short MNS data, we computed the evoked field at each sensor as the inter-trial average. With respect to the long MNS data, we subtracted the inter-trial average from all epochs in each dataset (separately for each participant and channel) to reduce the expression of the evoked response in the time-frequency plot, as analysis of these data focused on ERS and ERD.

**Figure 2:**
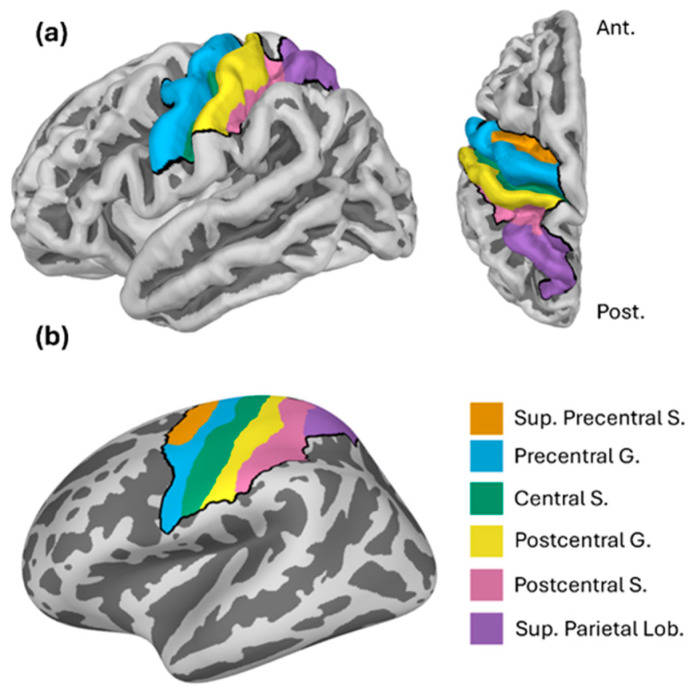
Anatomical regions of interest (ROIs) used for source localization. ROIs are shown on (**a**) a brain surface from lateral (**left**) and dorsal (**right**) views, and (**b**) an inflated surface. The dark border shows the area to which source localization was constrained. Abbreviations: Sup.: Superior; S.: sulcus; G.: gyrus; Lob.: Lobule.

We performed distributed source modeling on the evoked (short MNS) or epoched (long MNS) data from each task using the same procedure, described as follows. We computed a forward model and source-space grid for each participant, based on their scaled- template brain surface and BEM. We then applied minimum norm estimation to project the sensor-level data to the cortical surface. We constrained minimum norm estimation to a relatively large patch of cortex beneath our sensors, comprising a combination of the six regions of interests (ROIs) shown in Figure 2. Applying this constraint greatly reduced processing time and did not affect participant-level-evoked responses in these ROIs. These ROIs were: the superior precentral sulcus, precentral gyrus, central sulcus, postcentral gyrus, postcentral sulcus, and the superior parietal lobule (Figure 2). We selected these ROIs because of their proximity to our sensors, and because they have previously been used to investigate movement-related oscillatory changes [35]. ROIs were extracted from a standard anatomical parcellation [36] of the FreeSurfer template brain, morphed to each subject’s cortical surface. Source estimation returned a time course for every epoch/evoked field, at every vertex in the constrained source space. Finally, for each epoch/evoked response, we extracted one representative time course within each ROI as the first principal component of all composite source estimates.

We conducted time-frequency analyses on the long MNS datasets using the following procedure applied to sensor-level and source-estimated data. We computed time-frequency responses (TFRs) by applying Morlet wavelet analysis to the epoched data (3–40 Hz, 1 Hz steps, number of cycles equal to 1/3 of the center frequency). This produced one TFR array (time x frequency) per epoch and channel/ROI. We removed the first and last 0.2 s from each TFR array to eliminate edge artifacts induced by the wavelet analyses and averaged together the epoch TFRs for each participant and channel/ROI. Next, we baseline-corrected each TFR array by taking the logarithm of the ratio between power at each time point and mean baseline power, at each frequency (baseline was −1.0 to −0.5 s for long MNS). Finally, we averaged the baseline-corrected TFRs over the mu (8–15 Hz) and beta (15–30 Hz) frequency ranges, producing a mu and beta power change time course for every participant and channel/ROI. Grand-average TFRs and time courses were computed by averaging across participants.

We took a Bayesian approach to verify statistical evidence for SEF peaks and task-induced oscillatory changes relative to baseline amplitude/power. We computed Bayes factors BF_10_ which quantify relative support for the data under hypothesis 1 (H_1_, which predicts a difference from baseline) versus the null hypothesis (H_0_, no difference from baseline). Within this framework, BF_10_ values >1.0 and <1.0 signal support for the data under H_1_ and H_0_ respectively; the magnitude of BF_10_ in either direction (i.e., the degree of deviation from 1.0) signals the quantitative strength of evidence [37,38,39,40]. Bayes factors offer an alternative to conventional *p* values; they are informative as to quantitative strength of evidence (rather than merely rejecting H_0_), and do not require multiple comparison correction [38,41]. The following procedures were performed on source-localized time courses from each of our six ROIs. In the interests of maintaining reasonable scope, we do not report statistical analyses on the sensor-level results.

To quantify SEF peaks in the short MNS data, we defined narrow (3 ms) time windows to capture each peak in participants’ evoked fields for statistical testing. We first defined three a priori 10 ms windows, centered on 20, 35, and 60 ms following stimulation onset (broadly encompassing the expected N20m, P35m and P60m, respectively). Within each window, we then defined a smaller 3 ms window as ±1 ms around the peak latency in the grand-average (over subjects) evoked field. We also defined a baseline time window as −0.2 to −0.1 s. We then conducted one-tailed paired Bayesian *t*-tests to compare mean (over time) amplitude at the N20m, P35m, and P60m time windows to that of the baseline window (We did not have a priori predictions about the polarity of each peak in source space, since polarity depends on orientation of the underlying source and therefore will differ between ROIs. However, the polarity of the P35m and P60m should be opposite to that of the N20m in all ROIs. Therefore, we determined the direction - left- or right-sided - of our one-sampled *t*-tests based on the polarity of the N20m in the grand-average evoked field, independently for each ROI. For example, if the N20m was negative in a given ROI then we used a left-sided test to evaluate the N20m, and right-sided tests to evaluate the P35m and P60m. Using one-sided tests here ensured equivalent statistical stringency between the SEF analysis and the oscillatory analyses). Bayesian tests were implemented in the R environment using functions from the BayesFactor package (All *t*-tests used half-Cauchy JZS priors. JZS priors are default in the BayesFactor package [42]; the only change we made to default parameters was using a half-Cauchy, rather than full Cauchy, to enable one-tailed tests) [42]. Each *t*-test returned a Bayes factor BF_10_ wherein values > 1.0 could be interpreted as evidence for a difference in amplitude between the peak and baseline.

**Figure 3:**
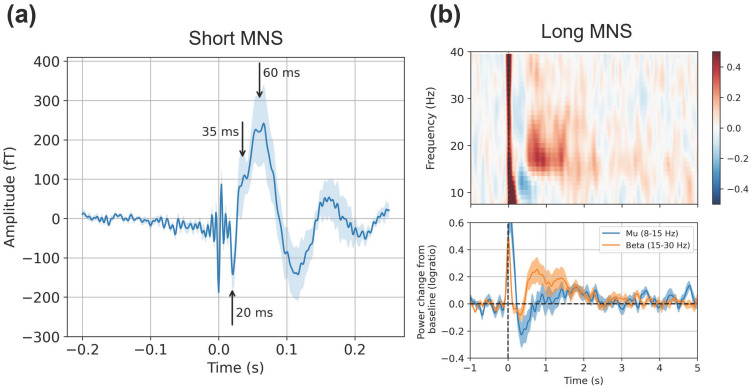
Grand-average results at one sensor (C3). (**a**) The evoked field elicited by the short MNS paradigm; (**b**) Mu and beta modulation elicited by the long MNS paradigm.

To quantify oscillatory changes (long MNS data only), we defined a priori time windows to capture baseline activity, ERD and ERS. The baseline period was defined as −1.0 to −0.5 s; the ERD period as 0.2 to 0.4 s; the ERS period as 0.5 to 1.0 s. The baseline and suppression window avoided the artifact induced by the MNS current. The suppression window was still centered on the beta ERD peak latency; however, since ERD follows stimulation (see Figure 3, for example) for each participant and dataset, we averaged the computed mu and beta time courses (see time-frequency analyses) over the baseline, ERD, and ERS time windows. This returned, per participant, a single value representing mean mu or beta power in each time window (with the exception that we did not compute mu power in the ERS periods, since there is no evidence for mu ERS in this context). To establish statistical support for mu/beta ERD, we conducted left-sided paired Bayesian *t*-tests to assess whether mean power during the ERD period was lower than mean power during the baseline period. For beta ERS, we conducted a right-sided *t*-test to assess whether mean power in the ERS period was higher than that of the baseline period.

For each participant, we generated maps indicating the sensitivity of our sensor layout to signals generated at each location on the cortical surface. Each map accounts for the sensor positions in the helmet, and the helmet-to-head and MRI-to-head transforms defined above. The sensitivity at each vertex is calculated by determining the vector norm of lead field (i.e., forward solution at the sensor locations) for a simulated 1.0 A-m source normal to the cortical surface adapted from mne.sensitivity map in [43]. In other words, we determined the expected sensor signal strength (in T) per unit of brain activity (in A-m) at each location in the brain. Each participant had a slightly different sensitivity profile due to the difference in head size and position in the helmet, leading to a different result per participant. We quantified ROI sensitivity as the average sensitivity across the constituent vertices. We used the grand-average sensitivity map (and ROI sensitivities) as a best estimate, and the standard deviation across participants to estimate variability. Grand-average source estimation results from the MEG data were considered in the context of the expected sensitivity discovered in this simulation.

We computed SNR around the N20m SEF peak at both the sensor and source levels. This enabled straightforward comparison of data quality in our MEG system to that of others, as previous estimates of SNR have been based on the N20m [3,4,11,44]. Moreover, the N20m was the most statistically robust response observed in our study (see Section 3); this measure is therefore reflective of maximal data quality attained from our system in its current state of development (e.g., field compensation is under development).

We computed SNR independently for each subject, sensor, and ROI as the absolute difference in mean amplitude between the baseline window and the (3 ms peak) N20m window, divided by the standard deviation of the baseline window. We then quantified sensor- and source-space SNR for each subject as the maximal value across sensors and ROIs respectively, similar to [44].

## 3. Results

**Figure 4:**
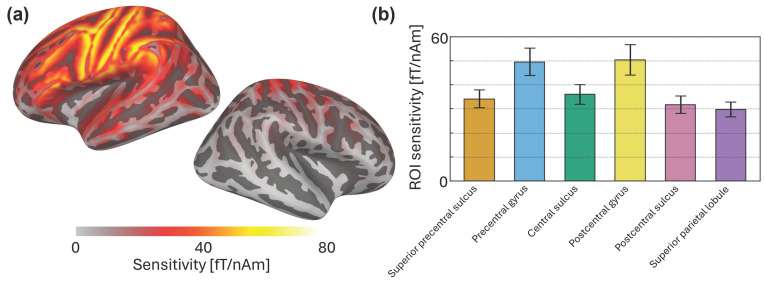
Grand-average results from the sensitivity analysis. (**a**) Simulated sensitivity to sources across the cortical surface. (**b**) Mean sensitivity in each ROI used for source estimation; error bars are ±1 SD across participants.

**Figure 5:**
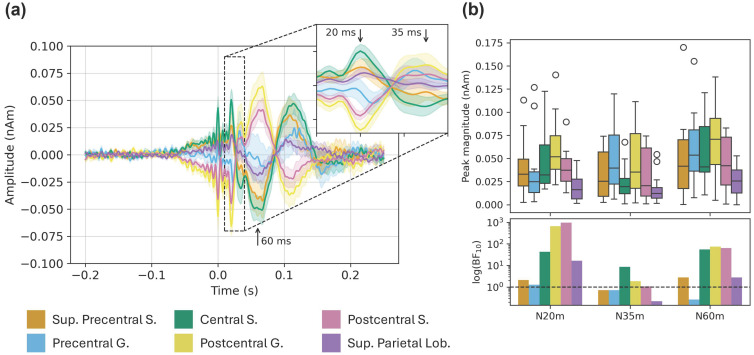
Source-localized SEF results. (**a**) Time courses show the grand-average evoked field in each ROI; ribbons are ±1 SE. (**b**) Top: Boxplots show magnitudes of the N20m, P35m, and P60m peaks (i.e., absolute amplitude difference between peak and baseline windows). Bottom: Bars show Bayes factors for each peak (note: logarithmic y-scale).

**Figure 6:**
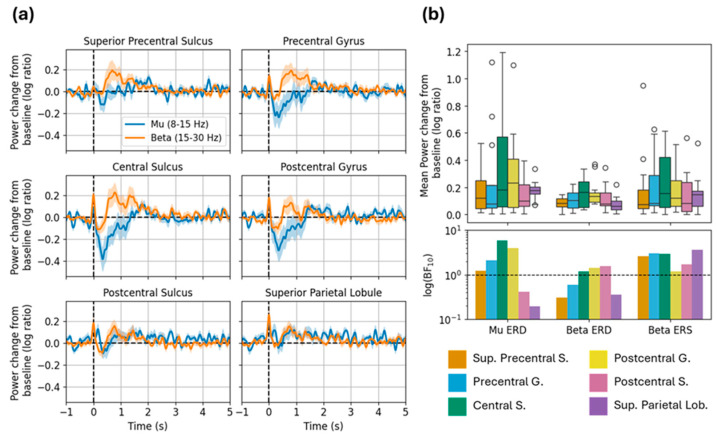
Source-localized mu and beta modulation in long MNS task. (**a**) Grand-average time courses show power change from baseline in each frequency band; ribbons are ±1 SE. (**b**) Top: Boxplots show mean power change from baseline within each window of interest (mu ERD, beta ERD, and beta ERS). Bottom: Bars show Bayes factors for each window of interest (logarithmic y-scale). Abbreviations: Sup.: Superior; S.: sulcus; G.: gyrus; Lob.: Lobule.

We investigated somatosensory-evoked fields (SEFs) elicited by an MNS paradigm with relatively short inter-stimulus intervals (labeled “short MNS”), and mu and beta modulation in data from an MNS paradigm with relatively long inter-stimulus intervals (labeled “long MNS”). We investigated these responses at both the sensor and source levels. Sensor-level results are shown in Figure 3. Sensitivity calculated in simulation is shown in Figure 4. Source-level results for the short and long MNS data are shown in Figure 5 and Figure 6 respectively, and results of statistical testing in source space (Bayes factors) are displayed in Table 1.

### 3.1. Sensor-Level Results

#### 3.1.1. Sensor-Level SEFs

The grand-average evoked field at one representative sensor (C3), shown in Figure 3a, exhibited the expected SEF complex. It comprised a clear negative peak at 20 ms (corresponding to the canonical N20m) followed by a broad positive deflection which briefly plateaued around 35 ms (P35m) and peaked at 60 ms (P60m). Note that the brief period of high-frequency deflections, roughly centered on 0 ms, is an artifact induced by the MNS current.

#### 3.1.2. Sensor-Level Mu and Beta Modulation

Figure 3b shows grand-average time-frequency responses (TFRs), as well as mu and beta time courses, also at sensor C3. We observed the expected suppression (ERD) in the mu and beta frequency ranges immediately following stimulation (with greater in magnitude in the mu range), followed by ERS in the beta range after around 0.5 s. As in the SEF data, artifact from the MNS current can be seen as a brief spike in power across all frequencies at 0 ms (the artifact is attenuated but not eliminated by subtraction of the evoked response).

### 3.2. Sensitivity Maps

The grand-average sensitivity map shown in Figure 4a demonstrates that the selected sensor layout was sufficient to capture neuromagnetic signals originating in the brain areas of interest for this study. When comparing across regions of interest (Figure 4b), sensitivity was maximal for sources in the pre- and post-central gyri. Sensitivity was lowest in the post-central sulus and superior parietal lobule, indicating that our sensor layout was not well adapted to capture activity from these areas. Thus, simulation findings align with our a priori expectation of sensitivity mainly to primary sensorimotor areas in this study.

### 3.3. Source-Level Results

#### 3.3.1. Source-Level SEFs

The grand-average SEF for each ROI in the short MNS paradigm is shown in Figure 5a. Here we saw clear peaks at 20 and 60 ms (and 35 ms to a lesser extent), particularly in the central and postcentral sulci, and the postcentral gyrus. Magnitudes for each peak were calculated as the absolute amplitude difference between each 3 ms time window and the baseline window (see Methods). With respect to the N20m and P60m: these peaks were maximal in the postcentral gyrus and decreased linearly in the anterior and posterior directions (Figure 5b). Results of our statistical analyses showed a similar trend: we saw the strongest evidence for the N20m and P60m (BF_10_ values around 600–1000 and 65–75, respectively) in the postcentral gyrus and sulcus, with strength of evidence decreasing in the anterior and posterior directions. These results are consistent with a neural generator in primary somatosensory cortex. Results for the P35m were more equivocal—statistical evidence for this peak was weak (compared with the N20m and P60m), and we did not see a clear spatial trend in peak magnitudes. We therefore consider the N20m and P60m to be robustly detected; the P35m less so.

#### 3.3.2. Source-Level Mu and Beta Modulation

In the long MNS data (Figure 6), the expected mu ERD and beta ERS effects were evident in multiple ROIs. Mu ERD was most evident in the central sulcus and postcentral gyrus; mean power change and strength of evidence was maximal in this ROI and decreased in the anterior and posterior directions. We saw a similar numerical trend with respect to beta ERD, though in this case the statistical evidence was relatively weak. Meanwhile, beta ERS appeared more anteriorly to the ERD effects. Broadly, we saw higher power change and strength of evidence in the superior precentral sulcus, precentral gyrus, and central sulcus compared to other ROIs, in accordance with expectation from prior literature and the sensitivity profile.

Notably, we observed the strongest evidence for beta ERS in the superior parietal lobule, despite the actual magnitude of this effect being relatively small (Figure 5b). This finding is unexpected (beta ERS should be maximal anterior to the central sulcus) and seems at odds with broader numerical and statistical trends described above. Furthermore, simulations indicate low sensitivity in this area. We return to this issue in the Discussion.

**Table 1:** Values are Bayes factors (BF_10_) for source-localized SEF peaks in the short MNS data, and mu/beta rebound peaks in the long MNS and button-press data. Abbreviations: Sup.: Superior; S.: sulcus; G.: gyrus; Lob.: Lobule.

ROI	Short MNS			Long MNS		
	N20m	N35m	N60m	Mu ERD	Beta ERD	Beta ERS
Sup. Precentral S.	2.13	0.71	2.81	1.21	0.3	2.56
Precentral G.	1.29	0.72	0.27	2.12	0.6	3.05
Central S.	42.53	8.54	55.34	5.9	1.18	2.97
Postcentral G.	657.14	1.86	75.37	3.9	1.44	1.17
Postcentral S.	964.62	1.07	64.16	0.42	1.55	1.71
Sup. Parietal Lob.	16.73	0.22	2.83	0.2	0.35	3.57

### 3.4. Signal-to-Noise Ratio

We computed sensor- and source-level SNR for the N20m, for each participant. We focused on the N20m due to the ubiquitous and robust nature of this response in MEG. At the sensor level, mean and median SNR across subjects was 6.73 and 5.23 respectively (range: 3.05–21.45), and we identified C3 as the most common sensor location at which SNR was maximal (C3 yielded the best SNR in 45.5% subjects). At the source level mean and median SNR were 8.67 and 8.66 (range: 1.22–25.87), indicating that source estimation did not substantially improve SNR—likely due to the small number of sensors. The central sulcus was the most common ROI at which SNR was maximal (33.33% of subjects).

### 3.5. Evaluating the Impact of HFC

HFC is typically applied to arrays with high channel counts and whole-head coverage; to our knowledge HFC has not been validated in partial-coverage arrays with low sensor counts. We therefore conducted a supplementary analysis to assess whether this step was appropriate for our data. We repeated our original analyses but this time omitted HFC during preprocessing. To maintain consistency between the two sets of analyses (with and without HFC), we removed the same manually identified bad channels and epochs during preprocessing. We compared results obtained with versus without HFC in terms of response magnitudes and SNR; quantitative outcomes of this comparison are briefly described here, with detailed results in Supplementary Materials.

At the sensor level, applying HFC dramatically reduced the magnitude of SEF responses and movement-related mu/beta modulation. This indicates that HFC subtracted a large proportion of brain signal from our data via lead field attenuation. This finding is consistent with previous work showing that HFC can cause substantial (~20–35%) lead field attenuation when applied to simulated single-axis sensors in a full-coverage array [34]. In our data, this reduction at the sensor level was closer to 50%, reflecting the diminished effectiveness of HFC in partial-coverage arrays. These effects were less pronounced at the source level, likely due to the effectiveness of source estimation at noise reduction. In source space, we saw a slight reduction in SEF magnitude across all ROIs, while oscillatory (mu/beta) power change from baseline appeared unaffected.

Despite the clear attenuation of neural responses induced by HFC (particularly at the sensor level), we found that performing analyses without HFC generally reduced SNR at both the sensor and source levels. For example, skipping HFC reduced the maximum SNR across ROIs by ~65% (median across subjects), and also shifted the location (channel/ROI) at which SNR was maximal towards posterior areas-away from central areas where the SEF is typically observed. These results suggest that, although HFC lead field attenuation removes a large component of signal generated by the brain, it was still marginally beneficial in that it removed relatively more noise from the recordings than true signal.

## 4. Discussion

In this study we sought to provide an honest evaluation of a low-channel-count OPM system, housed in a small cylindrical shield, for recording neuromagnetic responses in humans. Such systems are relatively inexpensive and straightforward to install and may provide an attractive alternative to current state-of-the-art systems (typically housed in MSRs and affording whole-head coverage) for researchers hoping to begin work in MEG. Considering recent enthusiasm for OPMs amongst the research community, it is important to properly characterize the utility and limitations of such systems. Here we focus on a commercially available system that may be of interest to other researchers considering similar setups. We remind the reader that active field compensation (i.e., “nulling coil” technology) was not implemented in this system at the time of this study. A bespoke field compensation for the cylindrical shield is under development—we hope to present its impact on performance in the future.

We investigated evoked and oscillatory sensorimotor responses (which are robustly detected by other OPM-MEG systems; see Section 1) following median nerve stimulation (MNS). These included the N20m, P35m, and P60m components of the somatosensory-evoked field (SEF) complex, and task-related modulation of mu and beta rhythms (beta and mu ERD; beta ERS). Furthermore, we assessed the spatial distribution of these effects on the cortical surface, as revealed by distributed source modeling, in the context of simulations of expected sensitivity for the given sensor layout. To facilitate direct comparison to previous MEG work, we also computed sensor- and source-level SNR around the N20m peak.

Broadly speaking, our sensor- and source-level results from the MNS paradigms were consistent with our predictions. With respect to the SEF complex, we identified clear peaks at 20 and 60 ms following stimulation, corresponding to the canonical N20m and P60m. At the source level these peaks were maximal (and accompanied by the strongest statistical support) in the postcentral gyrus and adjacent ROIs. This finding is consistent with previous localizations of SEF peaks to primary somatosensory cortex [12,13,14]. Our ability to isolate the P35m is likely limited by the relatively low sample rate and bandwidth of our system; most investigations of early SEF peaks acquire data at 1000 Hz with low-pass filtering at half the sample rate. We also observed the expected mu (and, to a lesser extent, beta) ERD immediately following stimulation, and a subsequent beta rebound (ERS) effect. At the source level, ERD and ERS were most evident in central-posterior and central-anterior ROIs respectively, again consistent with previous work [15,18]. These findings provide encouraging proof-of-principle support for our system. Not only could we detect these canonical responses, they also conformed to expected spatial distributions along the cortical surface. These results are consistent with previous work using a similar cylindrical-shield system and dual-axis sensors, which reported SEF and oscillatory sensorimotor responses at the sensor level [8], and successful localization of SEFs with dipole fitting [3,4].

We also computed signal-to-noise ratio (SNR) around the N20m peak for direct quantitative comparison to other MEG systems. Previous work has reported N20m SNR in the range of around 12–48 in SQUID-MEG systems [3,4,11,44]. With respect to OPM-MEG, previous work with partial-coverage arrays has reported SNRs around 42 at the source level [4] and 14–25 at the sensor level [8] (we are not aware of any studies reporting N20m SNR in whole-head OPM). By comparison, our system yielded relatively low SNR: around 7–8 (median) at both the sensor and source levels, with a maximum of 25.87 in one participant. We note that performing HFC (not applied in previous work with comparable partial-coverage systems [4,8]) did improve SNR overall, despite our sensors being arranged over a relatively small area, indicating that this preprocessing step can provide at least marginal gains when working with partial-coverage arrays.

We observed relatively low SNR compared to what is reported in other systems, likely due to a few contributing factors. First, our use of a small and passive shield likely provided less attenuation of environmental noise compared with conventional MSRs [4,11,44], which often benefit from additional layers of passive shielding and active field nulling. Indeed, Borna and colleagues [3] also employed active nulling in their cylindrical shield. Furthermore, a large MSR may provide a more homogenous field in the imaging volume (even without field nulling) due to the larger distance between the shield walls and the sensors. We hope to explore the impact of nulling in a cylindrical shield on MEG data quality in a future study. Moreover, as noted earlier, our system was limited to a sample rate of 1000 Hz. This is considerably lower than the sample rate of other investigations of the N20m (often ≥10 KHz, e.g., [3,4]). Given the temporal proximity of the stimulus artifact and N20m, the lower sample rate (and concomitant effect on the maximum low-pass filter frequency available) likely further impacted SNR. Finally, our limited head coverage, combined with our use of single-axis sensors, limited our ability to attenuate noise during preprocessing. In the context of source estimation, noise suppression tends to increase with channel count [45]. Similarly, homogenous field correction (HFC, an important noise reduction step applied during preprocessing) is more effective at separating signal from noise when applied to data with a plurality of sensor positions and orientations [34]. In light of these considerations, it is not too surprising that we observed relatively low SNR compared with other systems. Future improvements—in particular, incorporating multi-axis sensors and/or hardware capable of higher sampling rates—will likely yield SNR closer to that of other systems.

This work emphasizes the importance of simulated sensitivity analyses when working with partial-coverage systems. Sensitivity maps derived specifically for the participants’ head positions and sizes indicated that our sensor array appears to have provided good coverage of primary sensorimotor cortices (i.e., hypothesized regions of activation). This is confirmed by our experimental results, which show reasonable (given the system limitations) SNR to the responses of interest. For MEG users with limited sensor coverage, this highlights the value of simulations to ensure optimal sensor placement. Moreover, the sensitivity analysis provided important context for our results: consider the strange finding that the superior parietal lobule (SPL, an area not typically associated with sensorimotor processing) exhibited the strongest statistical evidence for beta ERS, despite very low source-estimated response amplitude and suspiciously low variance (markedly narrower error bars and boxplots compared with other ROIs in Figure 5b and Figure 6b). We can interpret the SPL findings as spurious because the sensitivity results indicate that our sensor layout (correctly) is not particularly sensitive to this area. This aligns with our a priori expectation, given that the sensor layout used in this study does not provide full coverage over the SPL, as shown in Figure 4. Thus, we posit that spurious findings are likely to occur without properly informed (i.e., guided by simulation) source estimation, particularly at the border of the sensor layout where there is asymmetric sampling of the lead field. For completeness, we also note that the sensitivity map confirms that we cannot reasonably report on ipsilateral hemisphere activation (e.g., weaker analogous oscillatory responses).

Here we evaluated a low-cost OPM-MEG system, comprising a low (16) number of single-axis sensors and a small cylindrical shield, for human neuromagnetic recording. This study yielded some important insights: the system in its current configuration appears sufficient to detect and localize canonical neuromagnetic responses in passive paradigms such as MNS. Moreover, modern preprocessing methods such as HFC, previously applied to data from systems with whole-head coverage, can improve SNR in partial-coverage systems such as this one. However, this must be assessed on a case-by-case basis dependent on the sensor positions and orientations and the signal(s) of interest. In general, researchers can expect higher HFC lead field attenuation (i.e., lost brain signal) with partial coverage, as compared to a full-coverage system. We noted markedly lower SNR to that seen in other contemporary systems, which presents a serious challenge for researchers interested in less robust neural phenomena. We encourage researchers to consider acquiring such a system to balance financial cost against desired applications and measurement sensitivity. The system tested here would definitely benefit from field compensation with current carrying coils, though this requires significant investment in engineering resources and development time, as well as further validation work (commercially available solutions are focused on planar shield walls, e.g., [46]). Incorporating more (ideally multi-axis) sensors and increased sampling rate will also likely improve SNR. We note that the post hoc sensitivity simulations confirm the importance of optimal placement of sensors to target a specific brain area when sensor count is low [24].

A major benefit of the system described here is that it requires considerably less mu-metal than one housed in an MSR. Mu-metal is both financially and environmentally costly, making up a large percentage of the purchase price of MEG systems. The cylindrical shield requires a surface area of around 12–13 m^2^ of mu-metal (2 walls, ~0.9 m diameter and ~2 m length, plus the end caps and a reducer). By contrast, a state-of-the-art “lightweight” MSR [46] requires around 84 m^2^ of shielding (Mu-metal square footage was estimated based on inner and outer volumes: 2.4 × 2.4 × 2.4 m and 2.8 × 3 × 2.8 m, respectively, reported in [46]). Therefore, a compact system such as ours offers a roughly six-fold reduction in mu-metal materials. Moreover, the shield could potentially be moved between environments. These features open the door to a range of applications. For example, OPM has shown promise for detecting and localizing focal epilepsy [47] and traumatic brain injury [48]; validation of movable systems could potentially improve clinical accessibility, particularly in the contexts of small community and mobile healthcare delivery.

## Data Availability

Data for this study is not publicly available, as participants did not consent to their data being made publicly available. Code for all analyses presented in this manuscript is publicly available on GitHub: https://github.com/lyambailey/OPM_MNSBP (accessed on 28 April 2026).

## Supplementary Materials

The following supporting information can be downloaded at: https://www.mdpi.com/article/10.3390/s26103131/s1, Figure S1: Grand-average SEF and mu/beta timecourses relative to MNS onset, obtained with HFC applied (left column; HFC ON) or omitted (right column, HFC OFF) during preprocessing; Table S1: Signal-to-noise ratio (SNR) obtained when HFC was applied during preprocessing (HFC ON) versus SNR obtained without HFC (HFC OFF).

## Abbreviations

The following abbreviations are used in this manuscript:

ERD	Event-related desynchronization
ERS	Event-related synchronization
MEG	Magnetoencephalography
MNS	Median nerve stimulation
MSR	Magnetically shielded room
OPM	Optically pumped magnetometer
SEF	Somatosensory-evoked field
SNR	Signal-to-noise ratio
SQUID	Superconducting quantum interference device

## References

[1] Brookes M.J., Leggett J., Rea M., Hill R.M., Holmes N., Boto E., Bowtell R. (2022). Magnetoencephalography with Optically Pumped Magnetometers (OPM-MEG): The next Generation of Functional Neuroimaging. Trends Neurosci..

[2] Fred A.L., Kumar S.N., Kumar Haridhas A., Ghosh S., Purushothaman Bhuvana H., Sim W.K.J., Vimalan V., Givo F.A.S., Jousmäki V., Padmanabhan P. (2022). A Brief Introduction to Magnetoencephalography (MEG) and Its Clinical Applications. Brain Sci..

[3] Borna A., Carter T.R., Colombo A.P., Jau Y.-Y., McKay J., Weisend M., Taulu S., Stephen J.M., Schwindt P.D.D. (2020). Non-Invasive Functional-Brain-Imaging with an OPM-Based Magnetoencephalography System. PLoS ONE.

[4] Boto E., Meyer S.S., Shah V., Alem O., Knappe S., Kruger P., Fromhold T.M., Lim M., Glover P.M., Morris P.G. (2017). A New Generation of Magnetoencephalography: Room Temperature Measurements Using Optically-Pumped Magnetometers. NeuroImage.

[5] Marhl U., Jodko-Władzińska A., Brühl R., Sander T., Jazbinšek V. (2022). Transforming and Comparing Data between Standard SQUID and OPM-MEG Systems. PLoS ONE.

[6] Rier L., Rhodes N., Pakenham D.O., Boto E., Holmes N., Hill R.M., Reina Rivero G., Shah V., Doyle C., Osborne J. (2024). Tracking the Neurodevelopmental Trajectory of Beta Band Oscillations with Optically Pumped Magnetometer-Based Magnetoencephalography. eLife.

[7] Bardouille T., Smith V., Vajda E., Leslie C.D., Holmes N. (2024). Noise Reduction and Localization Accuracy in a Mobile Magnetoencephalography System. Sensors.

[8] Borna A., Carter T.R., Goldberg J.D., Colombo A.P., Jau Y.-Y., Berry C., McKay J., Stephen J., Weisend M., Schwindt P.D.D. (2017). A 20-Channel Magnetoencephalography System Based on Optically Pumped Magnetometers. Phys. Med. Biol..

[9] Power L., Bardouille T., Ikeda K.M., Omisade A. (2024). Validation of On-Head OPM MEG for Language Laterality Assessment. Brain Topogr..

[10] Burgess R.C., Funke M.E., Bowyer S.M., Lewine J.D., Kirsch H.E., Bagić A.I., ACMEGS Clinical Practice Guideline (CPG) Committee (2011). American Clinical Magnetoencephalography Society Clinical Practice Guideline 2: Presurgical Functional Brain Mapping Using Magnetic Evoked Fields. J. Clin. Neurophysiol..

[11] Kimura T., Hashimoto I. (2001). Source of Somatosensory Primary Cortical Evoked Magnetic Fields (N20m) Elicited by Index Finger Stimulation Moves toward Mediolateral Direction in Area 3b in Man. Neurosci. Lett..

[12] Lin Y.-Y., Chen W.-T., Liao K.-K., Yeh T.-C., Wu Z.-A., Ho L.-T., Lee L.-S. (2005). Differential Generators for N20m and P35m Responses to Median Nerve Stimulation. NeuroImage.

[13] Ou W., Nissilä I., Radhakrishnan H., Boas D.A., Hämäläinen M.S., Franceschini M.A. (2009). Study of Neurovascular Coupling in Humans via Simultaneous Magnetoencephalography and Diffuse Optical Imaging Acquisition. Neuroimage.

[14] Wikström H., Huttunen J., Korvenoja A., Virtanen J., Salonen O., Aronen H., Ilmoniemi R.J. (1996). Effects of Interstimulus Interval on Somatosensory Evoked Magnetic Fields (SEFs): A Hypothesis Concerning SEF Generation at the Primary Sensorimotor Cortex. Electroencephalogr. Clin. Neurophysiol./Evoked Potentials Sect..

[15] Bardouille T., Bailey L., CamCAN Group (2019). Evidence for Age-Related Changes in Sensorimotor Neuromagnetic Responses during Cued Button Pressing in a Large Open-Access Dataset. NeuroImage.

[16] Byrne Á., Brookes M.J., Coombes S. (2017). A Mean Field Model for Movement Induced Changes in the Beta Rhythm. J. Comput. Neurosci..

[17] Houdayer E., Labyt E., Cassim F., Bourriez J.L., Derambure P. (2006). Relationship between Event-Related Beta Synchronization and Afferent Inputs: Analysis of Finger Movement and Peripheral Nerve Stimulations. Clin. Neurophysiol..

[18] Jurkiewicz M.T., Gaetz W.C., Bostan A.C., Cheyne D. (2006). Post-Movement Beta Rebound Is Generated in Motor Cortex: Evidence from Neuromagnetic Recordings. NeuroImage.

[19] Pfurtscheller G., Stancák A., Neuper C. (1996). Post-Movement Beta Synchronization. A Correlate of an Idling Motor Area?. Electroencephalogr. Clin. Neurophysiol..

[20] An N., Cao F., Li W., Wang W., Xu W., Wang C., Xiang M., Gao Y., Sui B., Liang A. (2022). Imaging Somatosensory Cortex Responses Measured by OPM-MEG: Variational Free Energy-Based Spatial Smoothing Estimation Approach. iScience.

[21] An N., Gao Z., Li W., Cao F., Wang W., Xu W., Wang C., Xiang M., Gao Y., Wang D. (2024). Source Localization Comparison and Combination of OPM-MEG and fMRI to Detect Sensorimotor Cortex Responses. Comput. Methods Programs Biomed..

[22] Hill R.M., Boto E., Rea M., Holmes N., Leggett J., Coles L.A., Papastavrou M., Everton S.K., Hunt B.A.E., Sims D. (2020). Multi-Channel Whole-Head OPM-MEG: Helmet Design and a Comparison with a Conventional System. NeuroImage.

[23] Schofield H., Hill R.M., Feys O., Holmes N., Osborne J., Doyle C., Bobela D., Corvilain P., Wens V., Rier L. (2024). A Novel, Robust, and Portable Platform for Magnetoencephalography Using Optically-Pumped Magnetometers. Imaging Neurosci..

[24] Hill R.M., Schofield H., Boto E., Rier L., Osborne J., Doyle C., Worcester F., Hayward T., Holmes N., Bowtell R. (2024). Optimising the Sensitivity of Optically-Pumped Magnetometer Magnetoencephalography to Gamma Band Electrophysiological Activity. Imaging Neurosci..

[25] Safar K., Vandewouw M.M., Rhodes N., Sato J., Taylor M.J. (2025). Mapping Neural Signatures of Face Processing in Young Children: An OPM-MEG Study. Soc. Cogn. Affect. Neurosci..

[26] Iivanainen J., Zetter R., Parkkonen L. (2020). Potential of On-Scalp MEG: Robust Detection of Human Visual Gamma-Band Responses. Hum. Brain Mapp..

[27] Oldfield R.C. (1971). The Assessment and Analysis of Handedness: The Edinburgh Inventory. Neuropsychologia.

[28] Bailey L.M., Bardouille T. (2025). Demonstrating the Need for Long Inter-Stimulus Intervals When Studying the Post-Movement Beta Rebound Following a Simple Button Press. Front. Neurosci..

[29] Pakenham D.O., Quinn A.J., Fry A., Francis S.T., Woolrich M.W., Brookes M.J., Mullinger K.J. (2020). Post-Stimulus Beta Responses Are Modulated by Task Duration. Neuroimage.

[30] Pfurtscheller G., Lopes da Silva F.H. (1999). Event-Related EEG/MEG Synchronization and Desynchronization: Basic Principles. Clin. Neurophysiol..

[31] Gramfort A., Luessi M., Larson E., Engemann D.A., Strohmeier D., Brodbeck C., Goj R., Jas M., Brooks T., Parkkonen L. (2013). MEG and EEG Data Analysis with MNE-Python. Front. Neurosci..

[32] Zhou Q.-Y., Park J., Koltun V. (2018). Open3D: A Modern Library for 3D Data Processing. arXiv.

[33] Hamalainen M.S., Sarvas J. (1989). Realistic Conductivity Geometry Model of the Human Head for Interpretation of Neuromagnetic Data. IEEE Trans. Biomed. Eng..

[34] Tierney T.M., Alexander N., Mellor S., Holmes N., Seymour R., O’Neill G.C., Maguire E.A., Barnes G.R. (2021). Modelling Optically Pumped Magnetometer Interference in MEG as a Spatially Homogeneous Magnetic Field. NeuroImage.

[35] Power L., Bardouille T. (2021). Age-related Trends in the Cortical Sources of Transient Beta Bursts during a Sensorimotor Task and Rest. NeuroImage.

[36] Destrieux C., Fischl B., Dale A., Halgren E. (2010). Automatic Parcellation of Human Cortical Gyri and Sulci Using Standard Anatomical Nomenclature. NeuroImage.

[37] Dienes Z. (2014). Using Bayes to Get the Most out of Non-Significant Results. Front. Psychol..

[38] Dienes Z. (2016). How Bayes Factors Change Scientific Practice. J. Math. Psychol..

[39] Lee M.D., Wagenmakers E.-J. (2014). Bayesian Cognitive Modeling: A Practical Course.

[40] Schmalz X., Biurrun Manresa J., Zhang L. (2021). What Is a Bayes Factor?. Psychol. Methods.

[41] Teichmann L., Moerel D., Baker C., Grootswagers T. (2022). An Empirically Driven Guide on Using Bayes Factors for M/EEG Decoding. Aperture Neuro.

[42] Morey R.D., Rouder J.N., Jamil T., Urbanek S., Forner K., Ly A. BayesFactor: Computation of Bayes Factors for Common Designs 2022. https://github.com/richarddmorey/bayesfactor.

[43] Gramfort A., Luessi M., Larson E., Engemann D.A., Strohmeier D., Brodbeck C., Parkkonen L., Hämäläinen M.S. (2014). MNE Software for Processing MEG and EEG Data. NeuroImage.

[44] Antonakakis M., Schrader S., Aydin Ü., Khan A., Gross J., Zervakis M., Rampp S., Wolters C.H. (2020). Inter-Subject Variability of Skull Conductivity and Thickness in Calibrated Realistic Head Models. NeuroImage.

[45] Vrba J., Robinson S.E., McCubbin J. (2004). How Many Channels Are Needed for MEG?. Neurol. Clin. Neurophysiol..

[46] Holmes N., Rea M., Chalmers J., Leggett J., Edwards L.J., Nell P., Pink S., Patel P., Wood J., Murby N. (2022). A Lightweight Magnetically Shielded Room with Active Shielding. Sci. Rep..

[47] Vivekananda U., Mellor S., Tierney T.M., Holmes N., Boto E., Leggett J., Roberts G., Hill R.M., Litvak V., Brookes M.J. (2020). Optically Pumped Magnetoencephalography in Epilepsy. Ann. Clin. Transl. Neurol..

[48] Dunkley B.T., Rajendram R., Preedy V.R., Patel V.B. (2022). Neural Activity and Oscillations as Biological Markers in Traumatic Brain Injury. Biomarkers in Trauma, Injury and Critical Care.

