# Supplementary material for: Neuromagnetism “On the Cheap”: Evaluating a Combined Cylindrical Shield and Partial-Coverage OPM-MEG System for Detecting Sensorimotor Responses in Humans
Source: Sensors (Basel). 2026 May 15;26(10):3131. doi: 10.3390/s26103131 (PMC13210914; doi:10.3390/s26103131)
Supplement: Supplementary file 1 [file sensors-26-03131-s001.zip › sensors-4180355-supplementary.pdf]

Supplementary Materials

Comparison of results with versus without HFC

We compared our main results - obtained with HFC applied during preprocessing - to those obtained without HFC. S Table 1 reports SNR values obtained from each method; S Figure 1 shows representative SEF and oscillatory (mu/beta modulation) responses at sensor and source levels.

Table S1. Signal-to-noise ratio (SNR) obtained when HFC was applied during preprocessing (HFC ON) versus SNR obtained without HFC (HFC OFF). As in the main results, SNR was captured as the maximum value across sensors or ROIs. Ranges and mean/median values across subjects (denoted by *) describe group-level measures of this metric.

Parameter	HFC ON	HFC OFF
Sensor Level		
Best channel	C3	CP3
% subjects for this channel	45.5	36.4
SNR range *	3.05-21.45	1.38-9.73
Mean SNR *	6.73	4.55
Median SNR *	5.23	5.23
Source Level		
Best ROI	Central sulcus	Postcentral sulcus
% subjects for this ROI	33.3	45.5
SNR range *	1.22-25.87	2.14-21.02
Mean SNR *	8.67	6.73
Median SNR *	8.66	5.23

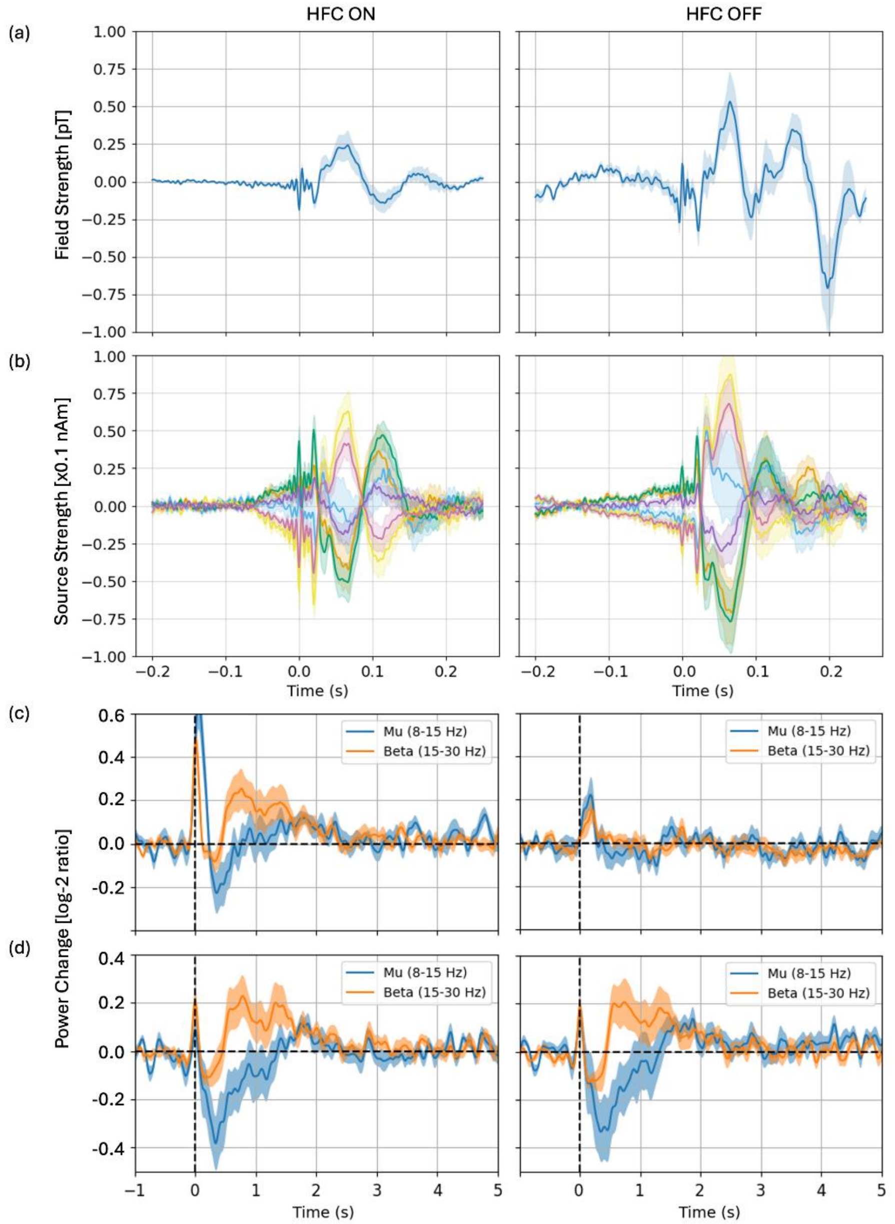

Figure S1. Grand-average SEF and mu/beta timecourses relative to MNS onset, obtained with HFC applied (left column; HFC ON) or omitted (right column, HFC OFF) during preprocessing. (**a**) Evoked response at sensor C3; (**b**) evoked responses at all ROIs, (**c**) mu/beta timecourses at sensor C3; (**d**) mu/beta timecourses at the central sulcus.
